# P03.02. From CAM practice to CAM research: a bridge fellowship for CAM practitioners to pursue careers in clinical research

**DOI:** 10.1186/1472-6882-12-S1-P255

**Published:** 2012-06-12

**Authors:** R Evans, G Bronfort, M Kreitzer

**Affiliations:** 1 Northwestern Health Sciences University, Bloomington, USA; 2 Center for Spirituality and Healing, Bloomington, USA

## Purpose

Patient oriented clinical research, particularly randomized clinical trials, is a complex field. Complementary and alternative medicine (CAM) educational institutions typically place less emphasis on research than what is typically seen in conventional university settings; consequently CAM practitioners are less prepared to pursue careers in clinical research than their conventional medical counterparts. The purpose of this presentation is to present a unique “bridge” fellowship that prepares CAM practitioners to pursue careers in CAM clinical research.

## Methods

Through a CAM Research Education Partnership Project (R25), Northwestern Health Sciences University (NWHSU), in collaboration with the University of Minnesota (UM), has designed a fellowship program which incorporates academic coursework, meaningful clinical research experiences, and one-on-one mentorship. Fellows enroll in the UM’s Masters of Science in Clinical Research program, where they receive formal research training in clinical trial methods, and have access to a wide variety of research training resources through the UM’s Center for Translational Research Institute. Hands-on research training occurs through a comprehensive program of multiple clinical research rotations at NWHSU’s clinical research center, where several CAM randomized clinical trials are underway. Individualized mentorship is provided by experienced, funded CAM and conventional scientists, who provide career guidance to fellows and involve them in all aspects of the conduct of CAM clinical research, including grant-writing, study design and implementation, grant management, team-building and leadership.

## Results

While the program is relatively new, it has begun to yield outcomes. Three CAM fellows (two chiropractors, one AOM practitioner) have been accepted and are enrolled in the UM’s Clinical Research Program. Collectively, they have authored several presentations and publications, and participated in the design and funding acquisition of two NCCAM funded projects.

## Conclusion

A fellowship program emphasizing coursework, practical training and mentorship may be effective in transitioning CAM practitioners into clinical research careers.

